# Mosaic Pattern of Lung Attenuation on Chest CT in Patients with Pulmonary Hypertension

**DOI:** 10.3390/diseases3030205

**Published:** 2015-09-07

**Authors:** Kamonpun Ussavarungsi, Augustine S. Lee, Charles D. Burger

**Affiliations:** Pulmonary and Critical Care Medicine, Mayo Clinic, 4500 San Pablo Road, Jacksonville, FL 32224, USA; E-Mails: Lee.Augustine@mayo.edu (A.S.L.); Burger.Charles@mayo.edu (C.D.B.)

**Keywords:** mosaic pattern, pulmonary hypertension, chest CT scan, pulmonary artery, screening

## Abstract

A mosaic pattern of lung attenuation on chest computed tomography (CT) may be due to various etiologies. There is limited published data on CT results when used to evaluate pulmonary hypertension (PH). We retrospectively studied the frequency of mosaic pattern in patients with PH and the cause of the PH by diagnostic group, as well as the correlation between the mosaic pattern and the following: demographics, severity of the PH, main pulmonary artery (PA) size, PA/aorta (PA/Ao) ratio, pulmonary function tests (PFT), and ventilation perfusion scan results. Overall, 18% of the cohort had CT mosaic pattern (34/189). Mosaic pattern was present in 17/113 (15%) in Group 1 pulmonary arterial hypertension, 5/13 (28%) in Group 2 pulmonary venous hypertension and 8/50 (16%) in Group 3 PH. Conversely, Group 4 chronic thromboembolic PH was more prevalent in 4/8 (50%). Main PA size, PA/Ao ratio, and segmental perfusion defect were positively associated with mosaic pattern. In contrast, factors such as age, gender, body mass index, functional class, hemodynamic data, and PFT values were not associated with mosaic pattern. Mosaic pattern is not specific as an isolated finding for distinguishing the subtype of PH.

## 1. Introduction

**Figure 1:**
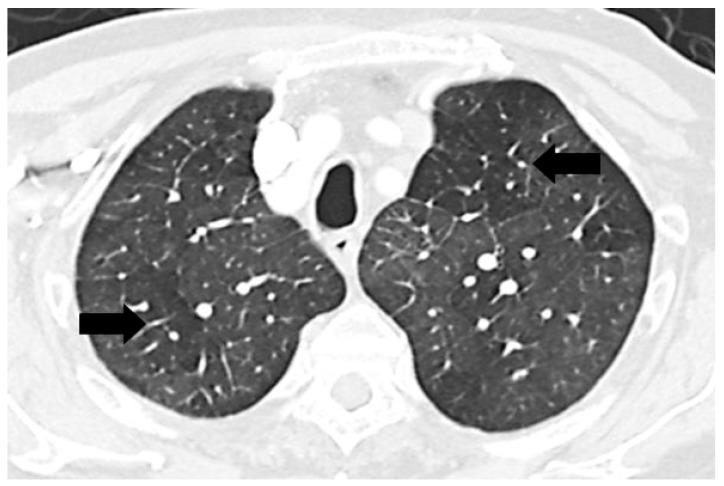
Chest computed tomographic (CT) scan demonstrates mosaic pattern of lung attenuation where there is a patchwork of regions of differing attenuation with well-defined borders (black arrows).

A mosaic pattern of lung attenuation on chest computed tomographic (CT) scan is defined by the Fleischner Society glossary as a patchwork of regions of differing attenuation seen on CT of the lungs [1]. It is characterized by heterogeneous lung attenuation with well-defined borders corresponding to the secondary pulmonary lobules as shown in Figure 1 [1,2]. This pattern is nonspecific and may be due to various etiologies, but the main pathologies are pulmonary vascular, small airway, and infiltrative lung disease [2,3,4,5,6].

The mosaic pattern related to pulmonary hypertension (PH) consists of relative hypoattenuation and hyperattenuation from adjacent areas with disparate perfusion [3,6,7]. The chest CT findings in patients with PH may also demonstrate enlargement of the main pulmonary artery (PA) with peripheral tapering [8,9,10,11]. The PA enlargement may be assessed by cross-sectional diameter or comparison to the diameter of the aorta (Ao), referred to as the PA/Ao ratio.

Chest CT scan is often utilized as part of the evaluation to define coexistent conditions related to PH [12]. Little is known regarding the frequency of the mosaic pattern in the PH population and factors predisposing to the presence of this pattern. We retrospectively examined the frequency of mosaic pattern among patients with PH and by cause of the PH (*i.e.*, group). We also sought to determine whether the presence of mosaic pattern attenuation on chest CT in patients with PH correlated with the severity of PH based on functional class and hemodynamic data from right heart catheterization (RHC), PA dimensions, pulmonary function test, and ventilation/perfusion lung scan.

## 2. Experimental Section

The study was approved by the Mayo Clinic Institutional Review Board. We retrospectively reviewed the patients referred to our PH Center from January 1992 to December 2006. Data was collected, including age, sex, body mass index, PH diagnosis group, modified New York Heart Association functional class, forced expiratory volume in 1 second/forced vital capacity ratio, total lung capacity, residual volume, ventilation/perfusion lung scan results, and hemodynamic data if the patients had RHC, including right atrial pressure (RAP), mean pulmonary arterial pressure (MPAP), pulmonary vascular resistance (PVR), cardiac output (CO). Patients who had either complete pulmonary function test or spirometry to review were defined to have obstructive airway disease if they had forced expiratory volume in 1 s/forced vital capacity ratio <70%, restrictive lung disease when total lung capacity <80%, and air trapping when residual volume >120%.

Of the 306 patients who were referred for PH evaluation, 117 patients without RHC were excluded. Chest CT scans on 189 patients had been personally reviewed by the one of the authors (CDB) who had evaluated the patients in the PH Center and noted whether a mosaic pattern was present in the physician notes in the medical record [1]. Those 189 patients served as the study cohort. Another author (KU) independently reviewed the imaging. For the correlation between PA dimensions and mosaic pattern evaluation, the original study was not available to provide sufficient cuts to determine the correlation of the PA diameter and mosaic pattern; therefore, there were only 162 in the secondary analysis. The main PA was measured at the widest short axis diameter in centimeters at the level of PA bifurcation on the axial section. The adjacent aorta was also measured and calculated for PA/Ao ratio.

All patients had undergone a clinical evaluation for PH. PH was defined as MPAP ≥ 25 mm Hg at rest by RHC. The patients with a PH diagnosis were clinically classified into five groups based on the 2013 World Symposium on PH [13]. Group 2 patients had PH related to left heart disease defined by a MPAP ≥ 25 mm Hg in association with a pulmonary capillary wedge pressure >15 and transpulmonary gradient <12 mm Hg [14].

Demographic data were described using descriptive statistics. Categorical data were described by frequency and percentage. Continuous data were presented as mean ± SD. Comparison between groups were assessed by chi-squaretests for categorical variables and student t tests for continuous variables. Mann-Whitney U-test was used if they were not normally distributed. *p* values < 0.05 were considered significant. All data were analyzed using SPSS version 16.0, Inc., Chicago, IL, USA.

## 3. Results and Discussion

We reviewed 189 patients with RHC who underwent a PH evaluation. The baseline demographic data of the patients are summarized in Table 1. Most patients were non-obese women. Most had functional class III–IV symptoms. More than half of the patients had Group 1 pulmonary arterial hypertension (PAH) with the second largest number of patients in Group 3 PH. All 189 patients were included for the presence or absence of mosaic pattern of lung attenuation [1] with a minority positive for this pattern. Overall, approximately 18% of the cohort had CT mosaic pattern as shown in Table 2. Mosaic pattern was seen in 17 of the 113 (15%) Group 1 PAH patients, 5 of the 18 (28%) Group 2 PVH, 8 of the 50 (16%) Group 3 PH and 4 of the 8 (50%) Group 4 chronic thromboembolic PH (CTEPH). Patients with Group 4 CTEPH were more likely to have mosaic pattern compared with other groups (50% *vs.* 17%, Fisher’s Exact test = 0.036 with the value of Phi at 0.175).

Chest CT scans in 162/189 (86%) were available for measurement of the diameter of the main PA and Ao. Overall, the mean diameter of the main PA was larger than normal [15,16] (3.45 ± 0.59 cm) with a normal Ao diameter (3.06 ± 0.42 cm) and resulting PA/Ao ratio of 1.14 ± 0.26. Patients with PH and mosaic pattern had significantly larger main PA size and PA/Ao ratio compared to the patients with PH and no mosaic pattern (Table 2). In contrast, factors such as age, gender, body mass index, functional class, hemodynamic data, and pulmonary function test results (available in 175 of 189 or 93%) were not associated with mosaic pattern. Comparison of the ventilation lung scans (available in 69 of 189 or 37%) /perfusion lung scans (available in 133 of 189 or 70%) demonstrated more segmental perfusion defects in the patients with mosaic pattern. Of note, all of those patients had Group 4 CTEPH. There was no statistical difference in delayed ventilation washout between patients with mosaic pattern and those without. Intraobserver variability in vascular dimension measurements showed high agreement with mean main PA difference at 0.3 mm (SD = 0.07, correlation at 0.98) and mean Ao difference at 0.5 mm. (SD = 0.05, correlation 0.97).

**Table 1:** Pulmonary Hypertension Demographics.

Variable	Total (N = 189)
Gender (% women)	124 (66%)
Age (mean ± SD)	60 ± 14
BMI (mean ± SD)	29.6 ± 7.6
WHO functional class	
1	7 (4%)
2	31 (16%)
3	113 (60%)
4	38 (20%)
PH diagnostic group	
1 Pulmonary arterial hypertension	113 (60%)
2 PH with left heart disease	18 (9.5%)
3 PH associated with lung disease and/or hypoxemia	50 (26.5%)
4 PH owing to chronic thrombotic and/or embolic disease	8 (4%)

BMI—body mass index; PH—pulmonary hypertension; WHO—World Health Organization.

**Table 2:** Summary statistics of demographic and clinical variables by mosaic pattern of lung attenuation on chest CT scan status.

Variables	Mosaic Pattern (*n* = 34)	Non-mosaic Pattern (*n* = 155)	*p*-value
Gender: Female	27 (80%)	97 (63%)	0.06
Age, year	57 ± 14	60 ± 15	0.28
BMI, kg/m^2^	28.6 ± 7.2	29.7 ± 7.8	0.55
PH diagnostic group					
1 Pulmonary arterial hypertension	17 (15%)	96 (85%)	0.25
2 PH with left heart disease	5 (28%)	13 (72%)	0.33
3 PH associated with lung disease and/or hypoxemia	8 (16%)	42 (84%)	0.83
4 PH owing to chronic thrombotic and/or embolic disease	4 (50%)	4 (50%)	0.03
Main pulmonary artery, mm	3.67 ± 0.7	3.39 ± 0.5	0.01
Aorta, mm	2.94 ± 0.3	3.08 ± 0.4	0.11
PA/Ao ratio	1.26 ± 0.3	1.11 ± 0.2	<0.01
Hemodynamic data, mean ± SD					
RAP, mmHg	11 ± 5.7	11 ± 5.5	0.45
MPAP, mmHg	46 ± 11.3	46 ± 14.3	0.73
CO, l/min	4.6 ± 1.7	4.9 ± 2.1	0.53
PVR, dyne s·cm^−5^	661 ± 419	605 ± 483	0.38
Pulmonary function test					
Obstruction (FEV1/FVC < 70%)	9 (27%)	49 (35%)	0.36
Restriction (TLC < 80%)	12 (36%)	58 (42%)	0.66
Air trapping (RV > 120)	5 (18%)	29 (23%)	0.52
Lung perfusion scan-Segmental defect	7 (21%)	8 (5%)	<0.01
Ventilation scan-Delayed defect	7 (21%)	29 (19%)	0.80

BMI—body mass index; PH—pulmonary hypertension; PA/Ao—pulmonary artery diameter/aorta diameter; RAP—right atrial pressure; MPAP—mean pulmonary arterial pressure; PVR—pulmonary vascular resistance; CO—cardiac output; FEV1/FVC—forced expiratory volume in 1 second/forced vital capacity; TLC—total lung capacity; RV—residual volume.

All patients had RHC. The mean RAP was 11 ± 6 and mean MPAP was 46 ± 14 mmHg, respectively. The mean CO was 4.9 ± 2.1 L/min with a calculated PVR of 615 ± 471 dyne s·cm^−5^.

In this single-center retrospective review, a mosaic pattern was observed on chest CT in approximately 1/5 patients evaluated for PH. The presence of mosaic attenuation in the setting of PH has been well described and is often attributed to Group 4 CTEPH but also has been reported in many PH conditions including idiopathic PAH and congenital heart disease [2,5,8,9,11,17]. The finding is explained by increased arterial vessel caliber in geographic areas of increased attenuation, or hyperemia, compared with decreased vessels in areas of low attenuation, or oligemia [8,9,10,11].

The purpose of our study was to examine the prevalence of the mosaic pattern in the various diagnostic groups of PH as defined during the 5th World Symposium on PH [13]. Our results suggest that a mosaic pattern is not specific enough to distinguish PH diagnostic groups because the pattern can be present in all PH diagnostic groups, although perhaps more prevalently in Group 4 CTEPH. The distribution across diagnostic groups did not appear to be influenced by demographics or PH severity as defined by functional class or hemodynamics (Table 2). Additionally, we could not determine the screening ability of mosaic pattern in PH due to the lack of a control cohort.

Published literature lacks the comparison among the PH diagnostic groups present in this study. For example, Sherrick *et al.* [5] retrospectively reviewed the frequency of mosaic pattern in 64 patients divided into three groups: PH due to lung disease (21 patients), cardiac disease (17 patients), and vascular disease (23 patients). Mosaic pattern was seen in 1/21 (5%) patients with PH due to lung disease, 2/17 (12%) with PH due to cardiac disease, and 17/23 (74%) with PH due to vascular disease. The latter group included pulmonary emboli, pulmonary veno-occlusive disease, lymphangitis metastasis and idiopathic PAH (four patients, two of which had mosaic pattern). By comparison, the present study demonstrated a lower frequency of mosaic pattern in Group 1 PAH; however, it had a different spectrum of subtype classification of PH and a larger cohort, with more confirmed diagnoses. Although we report a higher prevalence of mosaic pattern in Group 4 CTEPH, which is consistent with previous studies [8,11,17,18], the number of patients with CTEPH in our cohort is small. The time frame of the study predated the availability of pulmonary thromboendarterectomy at Mayo Clinic, Jacksonville, Florida; therefore, patients were typically referred to medical centers with that capability. A selection bias is highly likely as those patients often bypassed evaluation in the PH Center and were directed to centers offering surgical intervention.

In addition to examining the prevalence of mosaic attenuation in PH by diagnosis grouping, we sought to determine any correlation with the size of the main PA on chest CT, presence of obstructive or restrictive lung disease on pulmonary function tests, and corresponding abnormalities on ventilation/perfusion lung scanning. In particular, we were interested in correlative findings that might better elucidate mechanisms of the disparate attenuation in the lung parenchyma. For example, air trapping is a well-known mechanism of mosaic pattern on chest CT [2,3,4,5,6]; however, it did not seem to play a prominent role in this PH cohort (Table 2) as evidenced by the lack of prominence in Group 3 PH. Specifically, there was a similar prevalence of obstructive disease by pulmonary function test and air trapping by ventilation/perfusion lung scan.

Overall, the PH cohort in this study had larger than normal PA size and increased PA/Ao ratios. Normative values were based on two studies. The largest population cohort in which PA dimensions were assessed by CT, comes from the Framingham Heart Study, that reported a 90th percentile sex-specific cutoff value for main PA of 29 mm for men and 27 mm for women without cardiopulmonary risk factors [16]. The second study examined main PA diameters in 112 adult patients with documented normal MPAP by echocardiogram [15]. The mean PA diameter was 27 ± 2.8 mm in men and 25.9 ± 3 mm in women, with an upper limit of normal for the main PA considered to be 32.6 mm [15]. Both the mean values for both the PA size and PA/Ao were elevated in our study population. 

Furthermore, patients with PH and mosaic pattern had significantly larger main PA size and PA/Ao ratio compared to the PH patients without mosaic pattern (Table 2). As a mosaic pattern is felt to represent disparate pulmonary blood volume in adjacent lung segments, the areas with relative oligemia are felt to have more severe pulmonary vasculopathy [3,6,7]. One might hypothesize that the mosaic pattern may be indicative of more severe pulmonary vascular remodeling with increased vasoconstriction and PA narrowing. In addition, a reduction in PA compliance may produce more pronounced regional alterations in pulmonary arterial blood flow. The resulting increased PVR and larger upstream pressures might dilate the main PA; however, the lack of a correlation between mosaic pattern and hemodynamic data in this cohort argues against this hypothesis. More recent attention to ventriculoarterial coupling mechanisms with methods to access pulmonary impedance and PA distensibility [19] may offer opportunities for future research to understand the link between pulmonary vasculopathy and the upstream impact on the right ventricle and main PA. Nonetheless, the correlation of mosaic pattern with PA size and perfusion abnormalities suggests a link between heterogeneous small vessel disease and proximal PA dilation that may distinguish a clinical PH phenotype independent of diagnostic group and hemodynamic profile.

Our study had a number of limitations. Not every subject had pulmonary function tests, ventilation/perfusion lung scan, and CT scans available for second review.

Because the study was retrospective, the imaging protocol for the chest CT scan was variable depending on the clinical indication. Inspiratory and expiratory views were not performed routinely to evaluate the degree of air trapping. Lack of lung histology data also limited the actual diagnosis especially for Group 1 PH subtypes or Group 1' pulmonary veno-occlusive disease, but all patients were thoroughly evaluated and reviewed to ensure appropriate classification into the proper PH diagnostic group. The small sample size of each subgroup limits firm conclusions. The effect of PAH therapy and outcome were not evaluated in our study.

## 4. Conclusions

A mosaic pulmonary parenchymal pattern was observed on chest CT in approximately 1/5 patients evaluated for PH in our cohort. Analysis by the PH diagnostic group, revealed an increased prevalence in Group 4 CTEPH compared to the other groups. Regardless, mosaic attenuation is not specific as an isolated finding for distinguishing the subtype of PH. The correlation between mosaic pattern and main PA diameter, PA/Ao ratio, and segmental perfusion defect suggests a possible link between heterogeneous small vessel disease and proximal PA dilatation independent of PH diagnostic group or severity, a possibility that requires further study.
